# Local learning landscapes: conceptualising place-based professional learning by teachers and schools in decentralised education systems

**DOI:** 10.1007/s10833-024-09508-x

**Published:** 2024-07-04

**Authors:** Toby Greany, Tom Cowhitt, Andy Noyes, Cath Gripton, Georgina Hudson

**Affiliations:** 1 University of Nottingham, Nottingham, UK; 2 University of Glasgow, Glasgow, UK

**Keywords:** School leadership, Professional development, School autonomy, Place, Socio-spatial theory, Complexity theory, Networks, Organisational learning

## Abstract

This article sets out an original conceptual framework for place-based professional learning by teachers and schools in decentralised education systems. High quality Continuing Professional Development and Learning by teachers is associated with improvements in children’s outcomes. Most research in this area focuses on evaluating formal professional development programmes provided by external, non-school organisations. However, in practice, much professional learning is informal and takes place ‘on the job’. Meanwhile, in many systems globally, school leaders have been granted increased autonomy, for example taking on responsibility for the recruitment and professional development of staff. In these contexts, traditional place-based providers of professional development, such as Local Authorities and school districts, have been rolled back, while school leaders have been encouraged to draw on a wider marketplace of provision. These developments might create space for agency and innovation, but also present risks in terms of coherence, quality, and equity. For these reasons, we argue that there is a need to conceptualise the ways in which formal and informal learning occurs across complex local learning landscapes. We describe the iterative process through which the conceptual framework was developed before setting out the framework itself and the bodies of research and theory which underpin it. We draw on our empirical research using the framework in England to illustrate its three main contributions: as a heuristic device, an analytical tool, and an example of methodological innovation. We conclude by highlighting key implications for educational stakeholders, arguing that strengthening coherence, quality and equity across local learning landscapes in decentralised school systems requires attention to system governance and design as well as leadership and locality dynamics.

## Introduction

School systems around the world have been experiencing increasingly rapid change and reform efforts in recent decades (Mullis et al., 2016). Many governments are stepping back from hierarchical control of schools, adopting marketised and other New Public Management approaches as they seek to increase choice, improve quality, enhance equity and encourage innovation (Hood, 1991). One common thrust in all these approaches is to invest in support for Continuing Professional Development and Learning (CPDL) for teachers, given evidence that this is associated with improvements in the quality of teaching and, thereby, children’s outcomes (Cordingley et al., 2015; Darling-Hammond et al., 2017).

Teacher professional learning occurs both formally and informally. ‘Formal’ professional learning here refers to “structured, facilitated activity for teachers intended to increase their teaching ability” (Sims et al., 2021), including training courses and instructional coaching. ‘Informal’ learning here refers to a range of individual and collaborative activities and routines which enable professional learning, although these activities may also support wider outcomes (e.g. school improvement, lesson planning etc.). These informal activities range from conversations in the staff room or engagement in education-related reading and discussions (including via social media), through to more structured activities and routines, such as collaborative lesson planning, joint moderation of children’s work, lesson study, or engagement in subject networks. In the project report (Greany et al., 2023 :50–51) we map the most common professional learning activities reported by our interviewees onto a diagram structured on two dimensions—formal to informal and individual to collective. We heard that formal development, such as an externally run course, frequently provides a core focus for individual and collective learning. However, these formal activities invariably co-exist with a range of other modes of learning, some of which might be intentional and some of which occur ‘bottom up’ as busy professionals find ways to address their individual and collective problems of practice.

Most research in this area focuses on evaluating formal professional development programmes and interventions provided by external (i.e. non-school) organisations (Kennedy, 2019). In recent years several systematic and meta reviews have drawn this accumulating evidence on formal programmes and interventions together, often with the aim of identifying the features—or active ingredients—of effective professional development programmes and interventions (Sims et al., 2021; Fletcher-Wood and Zucollo, 2020; Kennedy, 2016). These efforts clearly offer important evidence to policy makers and designers of formal professional development programmes. However, we argue that a narrow focus on formal professional development programmes presents risks, given that, in practice, much—perhaps most—professional learning by teachers takes place informally and ‘on the job’, through professional conversations and a variety of more or less structured development activities, as outlined above (Hargreaves, 2010; Sebba et al., 2012). Furthermore, as the OECD (Boeskens et al., 2020: 30) recently observed:

Many countries [are seeing] an increasingly diverse set of CPL (continuing professional learning) providers, including third-party suppliers, competing for public funding and teachers’ resources (or, at a minimum, for their limited time). In many cases, teachers are drawing on a range of sources to access materials that support them in their self-directed learning, including online resources, discussion groups, videos, more traditional formats (e.g. guidebooks) and commercial training services. Likewise, schools may find themselves confronted with an increasingly extensive and *difficult to navigate* set of training options to support their teachers in their school-based CPL practices. (emphasis added)

These developments reflect wider changes stemming from New Public Management-inspired reforms in many school systems worldwide, including shifts towards school-based budgets and decision-making (OECD, 2011) and a parallel reduction in the role that ‘middle tier’ bodies, such as Local Authorities (LAs) and school districts, have traditionally played in more bureaucratic place-based systems (Cousin & Crossley-Holland, 2021; Greany, 2020; Lubienski, 2014). In more bureaucratic systems, LAs and school districts would generally co-ordinate the provision of CPDL opportunities for teachers and schools. In contrast, in decentralised systems, school leaders are frequently encouraged to draw on a wider marketplace of professional development provision, for example from commercial and non-profit providers (Steadman & Ellis, 2021; Boylan, 2023). Meanwhile, the rise of online learning and a variety of social media platforms for teachers have further accelerated these shifts, often making CPDL more available and bespoke, but also more complex (Perry et al., 2020a). 

As the OECD quote highlights, these developments can be ‘difficult to navigate’ for teachers and schools. We argue that this is by no means a peripheral issue. While the developments described above can certainly create space for agency and innovation in CPDL provision and present new possibilities for school and teacher engagement, they also present systemic risks in terms of (lack of) coherence, quality and equity. The importance of coherence in terms of how CPDL is structured and supported is clear in high performing school systems and districts globally, although such coherence can be achieved in different ways (Burns and Koster, 2016; Johnson et al., 2015). One example of this is Singapore, where schools are organized into geographical zones and clusters, with experienced principals serving as cluster superintendents and with two government funded providers working together to ensure coherent professional learning pathways for teachers and schools (Lee et al., 2021; Bautista et al., 2015; Dimmock & Tan, 2013).

For these reasons, we argue that the study of ‘local learning landscapes’ for teacher CPDL in decentralised school systems is an important but—as yet—under-studied area. The conceptual framework presented here grapples directly with these issues, seeking to identify the features that can make local professional development landscapes more or less ‘difficult to navigate’ and to combine this with wider insights into how professional learning develops within the messy reality of schools and policy-driven reforms. The framework was developed, tested, and iteratively refined through the process of conducting research into place-based professional learning by teachers and schools in England (Greany et al., 2023a). Informed by four areas of literature, the framework includes a set of six theoretically salient features which can be seen to interact in dynamic ways to shape how schools and teachers across a locality engage in formal and informal professional learning. Importantly, while the research in England *does* indicate that some local landscapes are more coherent, equitable, and high quality than others, the framework itself is not normative; it does not seek to define the features of a ‘good’ or ‘effective’ local learning landscape. Rather, it seeks to conceptualise the features and processes underpinning ‘local learning landscapes’, thereby making them more transparent and—potentially—amenable to influence by policy makers, researchers, and/or practitioners.

The article sets out the framework and the process through which it was developed, drawing on the underpinning literature and the empirical research in England. It is structured as follows. First, we outline the key contours of research into professional development and learning by teachers and schools, including in one specific curriculum area (mathematics). The literature on teacher professional learning is extensive, so we only summarise key tenets here by way of background. Next, we explore briefly the four bodies of literature (place, complexity theory, networks and organisational learning) that we drew on in developing the initial conceptual framework. We then describe the process of developing and testing the framework and outline the six constructs which make up the final iteration of the framework. The next section briefly outlines relevant recent developments in the English school system before drawing on the empirical research to identify what we see as the framework’s three main contributions: as a heuristic device, an analytical tool, and an example of methodological innovation. We conclude by discussing limitations and key implications for research, policy and practice in this area, arguing that strengthening coherence, quality and equity across local learning landscapes in decentralised systems requires attention to system governance, design and leadership as well as locality dynamics.

### Teacher professional learning

Most research into teacher professional learning focusses on formal professional development programmes and interventions, perhaps because these approaches are more straightforward to identify and evaluate (Kennedy, 2019). As we indicate above, in recent years, a series of systematic reviews and meta-analyses have synthesised findings from rigorous (i.e. experimental) studies, most of which have been conducted in the US, with a smaller number in Australia, the UK, and other European countries (Fletcher-Wood & Zuccollo, 2020; Kennedy, 2016; Sims et al., 2021; Timperley et al., 2007). This research indicates that well-designed and expertly facilitated programmes are associated with improvements in teaching quality and pupil outcomes. Furthermore, these studies have identified the features – or active ingredients—of effective programmes, albeit with some minor differences in view as a result of methodological debates (Higgins et al., 2018).

Evidence highlights the importance, and sometimes distinctive nature, of subject-specific professional development. For example, in mathematics – which was the particular curriculum lens for the study in England—research demonstrates the need for teachers to engage with personalised, career-long CPDL with a focus on developing their ‘Mathematical Knowledge for Teaching’ which includes continual development of both their knowledge about mathematics (subject matter) and about how to teach mathematics (i.e. ‘Pedagogical Content Knowledge’) (ACME, 2016; Heck et al., 2019). In addition, CPDL needs to develop teachers’ attitudes and beliefs (Guskey, 2002) as their prior experiences of mathematics mediate what they learn from CPDL (Ball, 1996) and many join the profession with a negative view of the subject (Hodgen & Askew, 2007). Mathematics CPDL that builds upon teachers’ current understanding and experiences, focusing on aspects of concern to individual teachers (Clarke, 2007), can make teaching more effective. These points highlight the need for school leaders—particularly curriculum and subject leaders—to understand where and how to contextualise CPDL to address the needs of specific subject areas and groups, including in mathematics (Cordingley et al., 2018).

These mathematics-specific CPDL findings begin to indicate some of the complexities involved, and to suggest why a narrow focus on formal programmes and interventions will be insufficient, because professional learning must engage with individual experiences, attitudes, and beliefs as well as subject and pedagogical knowledge, and is significantly shaped by the culture and context of the specific school in which a teacher works. These insights lead the OECD to conclude that:

The most promising path towards greater effectiveness in teachers’ CPL is not the mechanic compliance with a list of design features, but rather developing a better understanding of the way teachers learn, what motivates them to learn, and how, why and when specific design features can support this process. (Boeskens et al., 2020: 10-11)

Certainly, some researchers have sought to broaden the focus beyond formal interventions to consider how formal and informal learning can be conceptualised together and to encompass wider considerations, such as the role of school cultures, networks, and leadership in influencing teacher learning. For example, Darling-Hammond et al. (2017) included the need for coaching and expert support as well as collaboration, typically in job-embedded contexts, in their list of characteristics of effective professional development. Similarly, in a series of three reviews (Cordingley et al., 2015, 2018; 2020), Cordingley and her colleagues highlighted the need for teachers to have multiple, iterative work-based opportunities to incorporate new insights derived from formal inputs and to refine their practice informed by pupils’ responses, as well as the importance of active engagement by school-level leaders, including subject leaders. Cordingley et al.’s work also recognised that different schools will have different needs and different levels of capacity to engage with formal programmes, for example as a result of size or rurality. Other researchers have focussed on the role of communities of practice and professional learning networks in shaping practice and supporting formal and informal professional learning in education (Brown & Poortman, 2018; Schnellert, 2020; Vescio et al., 2008). This work all informs our analysis and addresses some of the same issues we tackle here, although as far as we are aware, no existing research has sought to conceptualise how these issues play out across local landscapes in the ways that we attempt here.

### Developing the framework

The local learning landscapes conceptual framework was developed by the authors as part of a two-year study of how primary schools (for children between four and eleven years) and teachers engage in mathematics CPDL across three localities in England (Greany, et al., 2023a). The six features included in the final version of the framework were distilled, iteratively, through the process of designing and conducting the research, as we explain below.

A starting point for the research was the decision to explore the CPDL landscape through the lens of place. This decision was largely motivated by evidence—described below—that England’s traditionally LA-co-ordinated school and professional development landscape had become fragmented in recent years as a result of marketisation and academisation. The research thus sought to explore how system and school leaders in three different localities navigate the “opaque complexity” of a “systemless system” (Lawn, 2013: 232), seeking to secure a locally coherent and equitable CPDL offer for schools and teachers despite varied values, capacities and increasingly diverse forms of school governance (Greany & Higham, 2018; Simkins & Woods, 2014).

Importantly, while the framework is informed by a review of literature and has been tested through empirical study, it is inherently connoisseurial, in the sense that it builds on our earlier research and experience and thus, inevitably, reflects our individual and collective interests, beliefs, and biases. Our positionality here includes that: two of us are mathematics education researchers while the other three might be described as policy and leadership scholars; some of us had researched professional development, place and/or complexity before, while others had not; and, overall, we share a commitment to undertaking rigorous, theoretically-informed research that also informs practice at conceptual as well as practical levels. One example of how our positionality has influenced this research is that we have all taught in schools in the past; this experience has arguably given us a practitioner perspective on the nature of professional learning which might, for example, have emphasised the importance of informal on-the-job learning more strongly than in other academic research.

We seek here to clarify what the framework is—and is not. We do not see it as a model—i.e. a template which captures a set of causal mechanisms that could be tested to predict the strength or effectiveness of local CPDL provision. Nor do we see it as a normative framework that describes what a local learning landscape ‘should’ look like. Rather, we see it as a heuristic device which can be drawn on to make important purposes, practices, processes, and people transparent in ways which might otherwise remain invisible. By positioning the framework in this way we recognise that it is ‘fuzzy’, by which we mean that it seeks to grapple with complexity and ambiguity in ways that some readers might find imprecise, but which we see as necessary given the range of contexts and issues that we are seeking to illuminate.

The process to develop the framework was highly iterative, but the main steps involved: (i) our research proposal to the Wellcome Trust (which funded the study) set out a preliminary conceptual framework (Appendix 1), based on our initial reading of the literature and previous research in this area; (ii) once the funding was confirmed and the project team recruited, a more detailed literature review was undertaken, focussed on the four areas outlined below; (iii) the first two meetings of the project advisory group (see Greany, et al., 2023a for membership) included in-depth discussion and feedback on the evolving framework and the associated literature review; (iv) in advance of our data collection in the three research localities, we developed an infographic setting out eight features of a ‘local learning system’ (Appendix 2)—this version of the framework was shared and discussed with interviewees throughout the data collection phase, including via a workshop in each locality at which emerging findings were interrogated; (v) through the final phase of data analysis and reporting, informed by three further meetings with the project advisory group, we distilled the initial eight features into the final six features set out below. During this final phase we shifted the metaphor from a ‘local learning system’ (Appendices 1 and 2), to a ‘local learning landscape’, as we felt this better captured the “systemless system” (Lawn, 2013) we had researched.

### Four bodies of literature

**Fig. 1:**
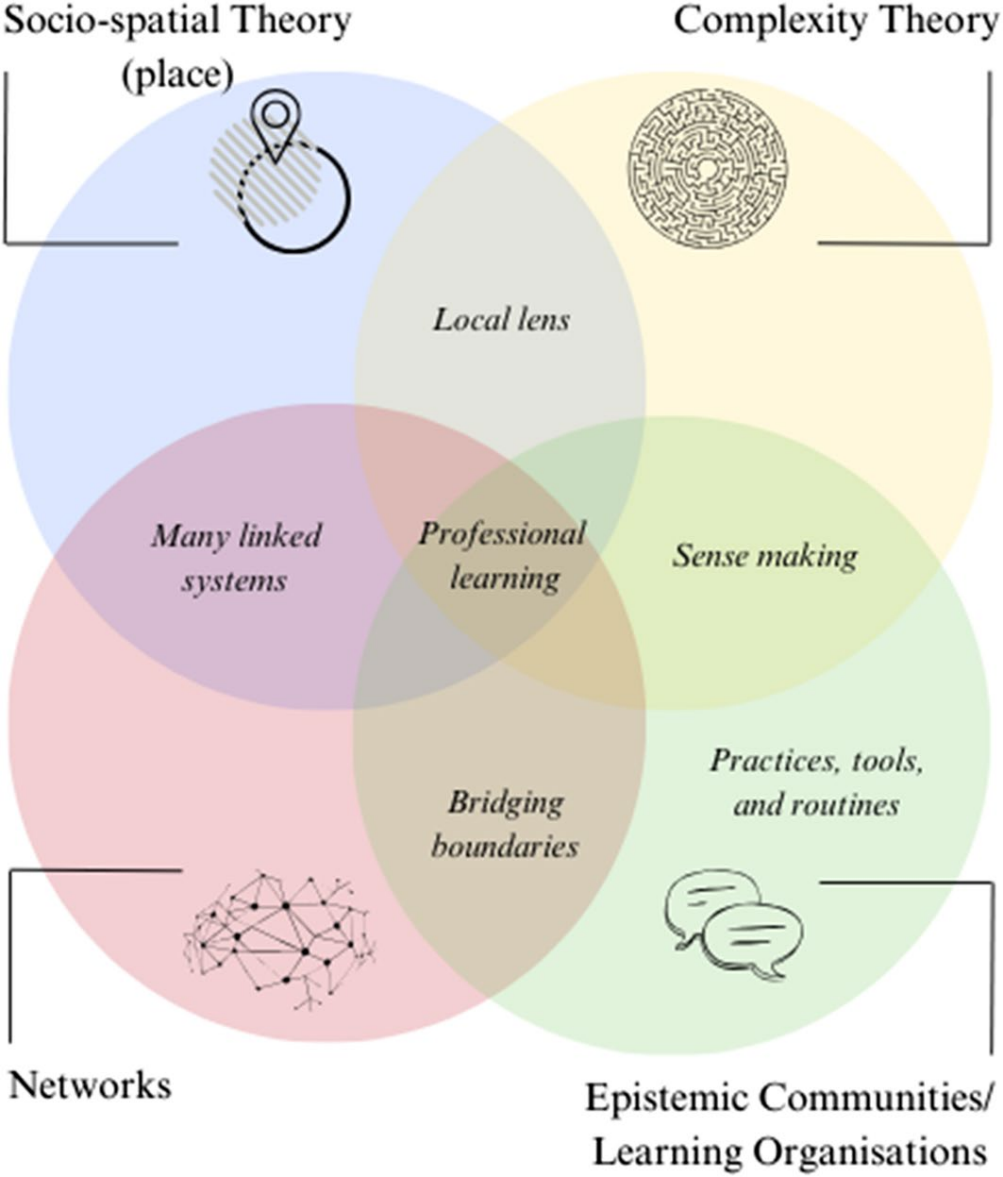
The four main (overlapping) bodies of literature drawn on in the literature review

**Fig. 2:**
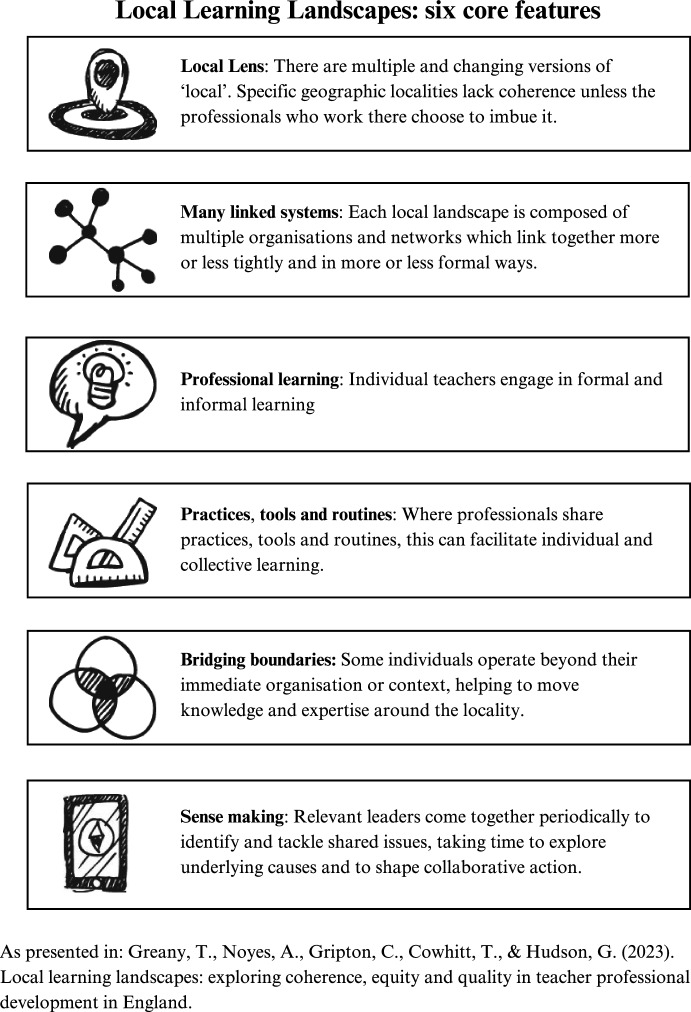
Local learning landscapes – six core features

Before introducing the framework, we outline the four main bodies of literature (Fig. 1) that we drew on in the literature review. We selected these particular bodies of research largely because they appeared relevant to our study research questions, which focussed on place/local, individual, and organisational learning, and the complex ways in which structures, processes, leadership/agency, networks, and relationships all shape this. At the same time, we acknowledge that we could have taken a different approach and that this selection was as much a reflection of our collective interests as a rational choice. We present these literatures as distinct but acknowledge that much of this work overlaps: for example, place and networks are described by Jessop et al., (2008: p389) as ‘mutually constitutive and relationally intertwined’, while the concept of ‘boundary spanners’, which we present here in the section on learning organisations, is also found in work on both networks and complexity (Williams, 2012). By showing the four bodies of literature as a Venn diagram, Fig. 1 seeks to recognise these overlaps and to show where the key concepts used in the local learning landscapes framework (Fig. 2) emerge from. Inevitably, there are sometimes overlaps with wider areas of literature which are not shown here: for example, we refer to isomorphism below in relation to networks but recognise that this concept is equally likely to be drawn on by neo-institutional scholars (DiMaggio & Powell, 1983).

The first body of literature was place—which is commonly explored alongside territories, scale, and networks in socio-spatial theory (Jessop et al., 2008)—reflecting the study focus on local landscapes for CPDL. The literature on place is vast and diverse, encompassing insights from geography, economics, sociology, and various other disciplines (Hubbard and Kitchen, 2011). While place has been utilised to some extent in educational research (Gorard et al., 2003; Gulson & Symes, 2007; Nespor, 1997; Thomson & Hall, 2016), as far as we are aware, it has not been applied to the study of CPDL. Four particular insights from this literature informed the conceptual framework, most obviously the ‘local lens’ feature. First, Cresswell (2004) sees place as a material location with distinctive features, a particular landscape and a unique identity, all of which are interconnected and “in movement through time” (Thomson and Hall, 2016: 15). Second, Massey (2005) highlights how these places are inherently porous and permeable, always connected—vertically, horizontally, through flows—to other places, ideas, things, and people. But places are not equal, they are shaped by particular power geometries, reflecting historic and contemporary social relations of class, gender, race, and disability. Furthermore, places are ‘thrown together’, unpredictable and messy: there can be no assumption of any singular coherence or identity. Third, Appadura (1996) recognises that localities—and the schools within them—are simultaneously ‘context derived’ and ‘context generative’; meaning that their ability to generate a distinctive local approach will be shaped by how (inter)national policies and norms impose standardised requirements, such as Ofsted (England’s national inspection body) school inspection grades in England. Fourth, a consideration of place and scale raises questions about vertical differentiation between ‘nested hierarchies’ (Jessop et al., 2008), which might include relations between a school, its LA or district, and any wider regional, state, or national governance arrangements.

The second body of research we drew on focussed on networks, which have also been studied from a range of perspectives—including at individual (Bidart, 2020; Kadushin, 2012) and inter-organisational levels (Popp et al., 2014). This includes a growing number of studies in education (see Greany & Kamp, 2022a for a detailed review), although none that we know of focus specifically on CPDL. Networks comprise relationships which allow for the exchange of material and non-material resources. Importantly, networks are inherently rhizomatic, meaning that they do not develop automatically or in consistent ways; rather, they are seen to operate along a set of dynamic continua, reflecting the strength, length, breadth, and depth of the relationships and activities that develop within and through the network (Perry et al., 2020a). Furthermore, networks commonly operate at multiple levels, often simultaneously, creating the potential for sub-networks and cliques which may or may not align to wider partnership goals (Townsend, 2015). Kadushin (2012) identifies three intrinsic needs which drive engagement in networks—safety, effectiveness, and status—arguing that different needs might be met by different types of network. In practice, network membership commonly reflects a tendency for homophily (‘birds of a feather’), while the process of collaboration in networks involves mutual influence (feedback), leading to a convergence in norms and behaviours over time (isomorphism). However, these tendencies can be problematic if they lead to exclusive cliques or prevent wider knowledge flows (Granovetter, 1973) and it is widely acknowledged that networks can have a ‘dark side’, for example if some schools are excluded (Grimaldi, 2011). Successful inter-organisational networks tend to display common features, including: a shared goal or interest that motivates collaborative action; shared commitment among network members, reflecting a degree of shared decision-making and a sense that benefits are shared equally; and shared values, practices, and attributes, such as reciprocity and trust. Networks tend to develop formalised governance and management structures over time and as they grow, believing this will improve efficiency, but such structures can risk reducing levels of ownership for (some) members (Provan & Kenis, 2008). Finally, leadership is widely recognised as a key ingredient in successful networks and a growing number of studies provide empirical evidence to support these claims (Sherer et al., 2021; Silvia & McGuire, 2010).

The third area is complexity theory, reflecting the inherently complex and ‘difficult to navigate’ CPDL landscapes that teachers and leaders must traverse in decentralised systems. Complexity has become increasingly popular among educational scholars in recent years (Greany & Kamp, 2022b; Jacobson et al., 2019; Boylan, 2018; Mason, 2016; Morrison, 2002). Complexity theories focus on sub-systems within systems and how these interrelate in influential ways, generating change. These systems include a range of actors: people, culture, nationality, community history, policy, funding (or lack of it), and so on. One implication is that research must move away from analysing individual entities and toward examining an ecosystem that is focused on, and arising from, a centre of interest: a “strange attractor” (Morrison, 2002: 324). Complexity theory thus provides several useful conceptual tools for making sense of local landscapes, but we focussed on three in particular. First is the concept of emergence, the “as-yet-unimagined” (Hager & Beckett, 2019; Jacobson et al., 2019) which reflects the “non-linear, unpredictable and generative” (Osberg & Biesta, 2010) ways in which complex entities respond to change. Second, feedback loops can influence complex systems; positive feedback typically generates growth, while negative feedback regulates and diminishes growth (Amagoh, 2016). Third, building on the points above on inter-organisational networks, the idea of many linked systems highlights how different organisations and networks are connected together in ways that may be more or less tightly coupled and more or less efficient (Hawkins & James, 2018).

The final body of work has two aspects: research on epistemic communities and learning organisations. The literature outlined above on place, networks and complexity is useful in making sense of complex, place-based change, but does not really address what makes a local landscape a *learning* landscape. In contrast, the mobilisation of knowledge and expertise through processes of learning are central in this final part of the review.

Epistemic communities—described as “knowledge-oriented work communities” (Holzner & Marx, 1979, 108)—provide a basis for practitioners to collaborate, even across different networks and organisational silos (Fleck, 1979; Haas, 1992; Kuhn, 2012). Epistemic communities can exist across large geographic areas (Hovey, 2005) or within smaller systems or organizational units. Critically, in an epistemic community, professionals adopt shared theories, language, and tools in order to construct, share, refine, and apply knowledge (Glazer & Peurach, 2015; Malone et al., 2021). Shared theory here refers to commonly held understandings within the community; for example, teachers might share implicit theories or rules of thumb in relation to what makes for a ‘good’ lesson. Shared language enables professionals to communicate in ways that go beyond everyday conversation. For example, an expert teacher will use specific technical language to dissect and discuss a lesson with a novice teacher. Members of epistemic communities will also draw on shared tools, such as a curriculum scheme, textbook, lesson planning template, lesson observation rubric, or an assessment framework. By adopting shared theories, language, and tools, professionals can communicate and collaborate more easily, without needing to constantly explain meanings or check understanding.

Finally, work on learning organizations highlights the need for “continuous learning for continuous improvement” (Watkins and Marsick, 2019). Specifically, schools and local schooling systems must develop capacities to navigate complexity and move knowledge and expertise around (Holmqvist, 2003; Zahra & George, 2002). Importantly, inter-organisational learning, for example between schools across a locality, requires a combination of strong within-organisational learning capacity combined with cross-organisational trust and knowledge-sharing processes (as outlined above in relation to networks). An organisation’s ability to exploit collaborative relationships thus depends on its own internal processes for sharing and using knowledge—known as its absorptive capacity*.* This absorptive capacity is tied to an array of organisational characteristics, such as the breadth of extant knowledge, structures, and roles that facilitate the sharing of knowledge within the organization (Cohen and Levinthal, 1990; Zahra & George, 2002). Boundary spanners, or individuals who are outward facing but still well-connected to local actors, can provide a bridge between different organisations, networks, and knowledge domains (Williams, 2012). These individuals often demonstrate “reticulist skills” (Bore & Wright, 2009, 243), as network builders, and are primary drivers of bricolage within systems by strategically working towards inter-organisational cohesion (Young & Eddy-Spicer, 2019). In professional networks, these important actors occupy structural holes, making them privy to diverse information and thereby putting them “at higher risk of having good ideas” if they seize the opportunity to synthesise different knowledges (Burt, 2004: 349). Sustained learning to support continuous improvement also requires sensemaking, through which school and system leaders work together to identify shared challenges, reflect on existing efforts to address these issues, and generate adaptive responses (Eddy-Spicer, 2019; Weick, 1995). Critically, sensemaking occurs through negotiated collective thinking in relation to ambiguous or ‘knotty’ issues, so is inherently interpersonal in nature, with school principals playing a key role in shaping such social process of meaning making (Coburn, 2005). These capacities help differentiate teaching as a profession by encouraging practitioners to adopt enquiry as stance, or a habit of mind that continuously challenges the status quo to ensure progress in quality and equity of education provision (Cochran-Smith and Lytle, 2015).

### The conceptual framework

Drawing on these literatures, and through the iterative process described above, a ‘final’ version of the framework was developed by the team. This comprises six features, shown in Fig. 2 below.

This final version of the framework does not pretend to draw together all the strands of literature and theory outlined above into a single, comprehensive set. Indeed, only a minority of the concepts referenced above are included. This reflects our aim to develop a framework that would be sufficiently simple and communicable to be interpreted and used by a range of busy policy makers and practitioners, most of whom would be unlikely to engage with a dense, concept and jargon heavy framework. Nevertheless, we argue that the framework is grounded in the theory outlined above, even where it is not explicitly named. A small number of concepts from the literature were included in earlier drafts of the framework, but then amalgamated or removed in the final version. For example, the first draft in Appendix 1 includes ‘Collective priorities and outcomes for Maths CPD’ as one element. This reflects the point that successful inter-organisational networks tend to have a shared goal or interest that motivates collaborative action. On reflection, we decided to remove this from subsequent versions (Appendix 2 and Fig. 2), we felt that it imposed an unhelpful normative and binary expectation—i.e. that a locality without ‘collective priorities’ could not be a ‘learning’ landscape. A much larger group of concepts were never directly included, but nonetheless informed our thinking and, in our view, underpin the framework, despite not being named. Table 1, above where we see these additional concepts broadly ‘fitting’, although this approach appears far more tidy than the reality, where many concepts straddle different parts of the framework. Table 1 includes concepts from the literature on professional learning (outlined above) as well as from the four areas of literature shown in Fig. 1 (with the relevant body of literature indicated in brackets for each concept).

**Table 1:** How concepts from the literature map onto the Local learning landscapes framework

Local lens	Place—a material location in movement through time (place) Power geometries—(in)equality, complexity, emergence, rhizome (complexity) Flows—porous, permeable, connected (place) Thrown together—coherence and identity not given (place)
Many linked systems	Scale—nested hierarchies (place/socio-spatial theory) Tight/loose coupling (learning organisations) Networks—motivations (safely, effectiveness and status); strength, length, breadth and depth of relationships and activities; shared values—reciprocity and trust; open/closed networks—homophily; governance and management structures; dark side—sub-networks, cliques (networks)
Professional learning	Formal and informal learning (professional learning) Developing subject and pedagogical knowledge, skills, practice (professional learning) Influence on attitudes, beliefs, and values (professional learning) School cultures and leadership influence teacher learning (professional learning) Organisational learning—absorptive capacity (learning organisations)
Practices, tools and routines	Shared theories, language, and tools (epistemic communities) Routines for constructing, sharing, refining and applying knowledge (learning organisations) Feedback loops and isomorphism (complexity)
Bridging boundaries	Reticulist—network leadership (networks) Knowledge mobilisation and structural holes (networks/learning organsiations) Professional expertise, trust and credibility (networks)
Sense making	Leadership reflection and data-informed evaluation to identify shared challenges and adaptive responses (learning organisations) Structures and agency—context derived and context generative (place) Emergence and ecosystem perspectives (complexity)

One reflection on Table 1 is that some of the framework headings, notably Local Lens and Many Linked Systems, are more concept-laden than others. This fits with our experience of conducting the study and analysis, where these two headings were particularly important in helping to unlock our understanding of each locality. This does not imply a hierarchy within the framework or that these two headings are more important, only that they help establish a core map, with the other four headings helping to illuminate the contours and implications of that map. A second reflection on Table 1 is that while most concepts from a single area of theory are clustered together within a single row (e.g. place under Local Lens, networks under Many Linked Systems etc.), some are not in the ‘obvious’ place. For example, we have put Appadura’s (1996) observation that places are simultaneously context derived and context generative into the column on ‘Sense making’. At one level we acknowledge that this is pure semantics and simply reflects the point above that some concepts straddle different columns (i.e. we could have put this under Local lens), but at the same we see it as an important strength of the framework that it draws on different theoretical traditions and mixes them together in fuzzy ways that can shed new light on issues. We return to this point below.

In the next section we draw on the research in England to exemplify what we see as the framework’s three main contributions. This includes examples of how we drew on the framework to support our analysis in England. These examples begin to show how the framework can be used in practice and how the six features can be seen to interact with each other. In developing and applying the framework we found that the six features often operate in pairs:

- ‘local lens’ and ‘many linked systems’ work together to support an assessment of core identities and structural arrangements for professional development across a locality;
- a focus on ‘professional learning’ and ‘practices, tools and routines’ reveals how formal and informal learning opportunities operate and how shared (or not) practices, tools and routines support this;
- finally, ‘bridging boundaries’ and ‘sense making’ help highlight the role of key individuals and governance processes in shaping professional learning.

That said, we do not want to overplay these couplings or to prescribe a single way of applying the framework. Rather, we argue that each of the six areas can be seen to interact with each other and that this multi-layered fluidity is necessary for nuance and complexity to be teased out. Indeed, when we shared our emerging research findings with participants in each of the three localities (at that point using the eight features from the draft framework in Appendix 2), we deliberately showed them visually in a non-hierarchical format, with multiple linkages between features (for an example from one locality see Appendix 3).

### Exemplifying the framework in practice – evidence from England

This section draws selectively on the local learning landscapes research in England to support what we see as three main contributions that the framework makes to research, policy and practice: i) a heuristic device which refocuses attention on professional learning in localities; ii) an analytical tool for identifying purposes, practices, processes, and people as well as associated risks and issues within or across localities; and iii) an example of methodological innovation. In order to set the context for international readers it starts by briefly outlining recent developments in England’s schooling and CPDL landscape. We provide several illustrative examples of the framework in practice below but encourage readers to review the project report (Greany, et al., 2023a) for an in-depth treatment.

England’s 21,000 publicly funded schools have been relatively autonomous by international standards for several decades (OECD, 2011). However, until 2010, its 152 Local Authorities retained a significant role in overseeing most schools and co-ordinating a local (i.e. place-based) CPDL offer and approach. This changed in the years after 2010 as a result of the ‘self-improving, school-led’ system reforms pursued by a succession of Conservative-led (centre right) governments (Greany & Higham, 2018). This agenda saw the rapid expansion of academy schools (akin to charter schools in the US); academies are non-profit companies that are funded and overseen by national rather than local government, so their expansion has led to a significant reduction in the capacity and role of LAs (Greany, 2020). Over a decade on, more than a third of all primary schools and four in five secondary schools have become academies. These academies can operate as single stand-alone schools, but most are part of a Multi-Academy Trust (MAT). There are currently more than 1200 MATs in England, operating anywhere between two and 50+ academies within a single organisational structure overseen by a board and Chief Executive (Greany & McGinity, 2021). MATs are responsible for the quality of schools they oversee. In general, the MAT will have an internal CPDL strategy and approach, meaning that a school within a MAT will access much of its CPDL from within its trust, although there are important differences between how different MATs operate, for example as a result of size (Greany, 2018). Importantly, MATs are not place-based, meaning they can operate schools across any geographic footprint, and it is increasingly clear that ‘local’ partnerships between schools are becoming fragmented as schools in the same geographic area join different MATs (Greany et al., 2023b).

In terms of professional learning, the policy focus in the years after 2010 was on encouraging ‘school-led’ provision. Around 750 high performing schools were designated as ‘teaching schools’ and given a remit to provide CPDL and improvement support to other schools, thereby plugging some of the gaps in provision left by the roll-back of LAs (Greany & Armstrong, 2022). However, over time, research showed that rural and remote areas of the country were poorly served by this model, and that the schools which could benefit most from CPDL were often less likely to engage for reasons of cost and capacity (Ovenden, Hope and Passey, 2019). In response, since around 2016, the government has moved away from the patchwork of ‘school-led’ provision, towards a nationally defined career-long framework delivered via a range of approved providers and school-based curriculum hubs. Many of these CPDL hubs are based in larger MATs, but with a remit to provide support to all schools that want it across a defined geographic footprint (much larger than the previous LAs). Each hub focusses on a specific area of the curriculum (English, Maths, Computing, Modern Languages etc.) or an aspect of school improvement (teacher and leadership training, research use, behaviour management etc.), but these schemes have been developed at different times and with different criteria, levels of resource and footprints – meaning that the landscape of hub provision is hard to navigate for schools.

We turn now to the local learning landscapes research, using examples from the study to draw out what we see as its three main contributions. The research itself focussed on primary teacher and school engagement with mathematics CPDL across three diverse localities in England—City, Town and Shire. In total, 82 teachers, subject leaders, school leaders and system informants were interviewed, and 19 school case studies were completed. The research received ethics approval from the University of Nottingham School of Education Ethics Committee. For the detailed methodology and a discussion of some of the challenges involved in undertaking place-based research in education see the project report (Greany, et al., 2023a; Gripton, et al., 2022).

The framework’s first contribution is as a heuristic device which allows policymakers, practitioners, and researchers to ‘see’—or perhaps ‘refocus on’—the local learning landscape. We argue that in England the focus on locality has largely been lost in recent years, due to the roll-back of LAs as place-based coordinators of education, the rise of non-place-based MATs, and the roll out of nationally developed, formal professional development programmes and initiatives. As a result, policy and practice had arguably become myopic—it was simply too hard for busy professionals to step back and ‘see’ the ways in which local collaboration and informal learning between schools and teachers was becoming more difficult in a context of fragmentation and balkanisation. In many ways, this myopia is understandable given the sheer range, scale, and complexity of the issues and contexts at play. By recentring the local and informal in all its messy complexity, and by making these issues visible in a simple format, the framework goes a small way towards correcting the myopia, thereby offering scope for collective review and sensemaking. We have various examples of this heuristic refocussing role playing out in practice; for example, one research interviewee (who was responsible for a local CPDL hub) asked if the draft framework (Appendix 2) was copyrighted and whether he could use it to train his hub team, while another locality leader (who was not involved in the research, but who attended the project launch event) has subsequently used the framework as the basis for a new local learning strategy which brings together the various hubs and other CPDL actors across their area.

The second contribution builds on the first but highlights its potential as an analytic tool which can be used by policy makers and practitioners to assess both strengths and areas for development in relation to CPDL in any given locality. It does this by—implicitly—raising questions about the purposes, practices, processes, and people that either currently do, or potentially could, influence levels of coherence, quality, and equity in the local learning landscape. By using the framework as an analytic tool, through a process of collective dialogue and sense-making involving local stakeholders, policy makers and practitioners could identify risks and issues in their current approach as well as potential areas for attention and development. There are numerous examples of how the framework supports such analysis from the research in England but we focus on three here.

The first came from the focus on ‘practices, tools and routines’, informed by work on epistemic communities, which revealed how different schools and MATs in each locality were developing distinctive approaches to ‘mastery’ in the teaching of mathematics. These differences came partly from the influence of competing commercial CPDL providers, each working to differentiate and sell their ‘mastery’ products, and partly from the lack of coupling (i.e. ‘Many linked systems’) between schools and MATs in all three localities (but particularly Town). As one local system leader explained:

*The challenges we've got as well, with more schools moving in to Multi Academy Trusts, is they like to do their own in-house PD. Uh, you know. So if you've got a… (MAT) with, you know, six secondary schools in (locality name) they like to keep all PD in house, you know? So you have got that shared language, that shared understanding of teaching and learning that is shared within that trust. (System leader, Shire)*

This finding highlights a need for policymakers and practitioners to consider how they might ameliorate the negative impact of markets and fragmentation in CPDL provision.

A second example was in how the framework revealed the presence or absence of local boundary spanners in all three localities; we refer to these actors as ‘landscape gardeners’. These individuals possessed multiple different job titles and were deployed in different professional roles across a school system. All demonstrated reticulist network-building skills, helping to connect different schools, MATs, and CPDL providers, but the value they added went beyond serving as the connective tissue—or glue—in otherwise fragmented local landscapes. They were also experienced and locally credible educators, who were privy to different types of information as a result of their varied careers and, often, portfolio roles, making them ideal candidates for developing innovative solutions to enduring challenges within the system. However, because these ‘landscape gardeners’ had roles that were often unstructured (many worked part-time in two or more different organisations – such as a school and a curriculum hub) they were relatively invisible and therefore unsupported and often under-valued. In Town an established ‘landscape gardener’ had recently left to work elsewhere as a result of changes in local hub designations, leading to a breakdown in local relationships. This raises questions about how such ‘landscape gardening’ work could be structured, recognised, and rewarded in future.

A third example of the framework’s analytical purchase is how it highlighted the lack of ‘sensemaking’ fora and structures in all three localities. Following the roll-back of LAs, no one organisation or group has responsibility or accountability for looking across the locality to identify needs, strengths, or areas for development in relation to CPDL, thereby potentially missing opportunities to enhance coherence, quality, and equity on behalf of all children. Translating new policy within local contexts is critical as policy implementation “works by accretion and sedimentation rather than revolution” (Ball, 2021: 63). Incoherence and gaps in provision can occur in times of rapid transition, especially with the encouragement of new CPDL providers and the rapid expansion of online provision following the pandemic (Lubienski, 2014). This was clearly evident in England, where our research took place during the introduction of Teaching School Hubs. Eighty-seven of these new hubs were tasked by government with taking over many of the responsibilities of the former teaching schools (of which there were 750), such as overseeing school-based initial teacher training and delivering specialist professional qualifications. However, the dissolution of teaching schools also resulted in the collapse of many existing networks and routines which had helped to facilitate inter-school collaboration and professional learning (Greany & Armstrong, 2022). The framework thus helped to identify the importance of incumbent local actors who could hold on to significant institutional memory and relationships, drawing these back together to provide a level of coherence during rapid policy transitions. This was most apparent in City, where a small group of ‘landscape gardeners’ had worked over many years to submit ‘collaborative bids’ which brought together local MATs, (former and newly designated) hubs and wider CPDL providers into relatively coherent ‘many linked systems’. Nevertheless, even in City, a senior leader from the local authority reflected on how incoherence was now baked into the local landscape, making strategic sense making all but impossible:

*We don't have fairly regular conversations as [name] local authority with the Maths Hub actually. So that's just made me reflect on, you know, Why? Why that is the case? Because if we were to be a bit more systematic and systemic in our thinking around mathematics development, surely we need to have regular meetings with the leaders of the Maths Hub in order to know which schools were engaging, what they're engaging with and what the impact of that is being. But we, I, don't have sight of those metrics if I'm honest with you. (System leader, City)*

The framework’s third, more modest, contribution is methodological. Wrestling with complexity is part and parcel of conducting research in the social sciences, but we would argue that researching local learning landscapes presents a particularly ‘wicked’ problem (Rittel and Weber, 1973). We are not the first to observe that contemporary education can neither be clearly understood nor resolved by a single actor. This is certainly true of local CPDL landscapes, particularly in decentralised and fragmented contexts such as England. One challenge is the need for scale jumping and the interactions between scales in complex eco-systems: from the teacher, to the school, to the MAT, curriculum hub or LA, to the national, international and online forces that shape contemporary education. One of us has written about this challenge before (Noyes, 2004; 2013), arguing that mixed methods designs offer the potential to ‘zoom in’ and ‘zoom out’, from the individual case study to the statistical analysis of system-level outcomes over time. As we have outlined in this paper, in the local learning landscapes study we adopted a different approach, by developing a theoretically-informed initial framework which was then tested and refined through a process of qualitative research. Of course, we are not the first researchers to have done this, but we note that research on school improvement and professional learning has long been critiqued for being largely atheoretical (Kyriakides et al., 2020; Scheerens, 2013; Trujillo, 2013). Thomson and Heffernan (2021) suggest four ways in which theory gets used in educational research: (i) the framework grows organically with the research; (ii) you arrive at the framework somewhere in the middle of the project; (iii) the theoretical framework is developed at the outset of the project; (iv) the theory is developed towards the end of the field work. The approach presented here seems to straddle all four of these options, perhaps indicating a degree of innovation. One particular benefit of the approach in our experience was that it helped to meld our thinking as a research team, given our diverse backgrounds as outlined above. By developing an original framework and by refining it together, we benefitted from and were often able to integrate these diverse experiences in ways that might not otherwise have been possible. Our experience in other projects has sometimes been that in order to satisfy funding requirements, the research—including any conceptual framework—is overly specified in advance. We thus encourage researchers and funders to consider how conceptual frameworks, such as this one, can be used more developmentally.

## Limitations

Without doubt, the framework has limitations. We imply above that our decision to grapple with individual, organisational and inter-organisational learning, and the interactions between them, and to draw four different areas of research and theory into a single, accessible framework, could be seen as over-ambitious at best, and unhelpfully imprecise at worst. We certainly acknowledge that the work is conceptually ambitious, but our experience of sharing it with policy makers and practitioners in England suggests that it is nonetheless helpful and that they ‘recognise’ the concepts and issues addressed. Indeed, following a recent presentation, we were contacted by a local hub leader who exclaimed ‘finally, now I’m nearly at retirement, I know what my job is – I’m a landscape gardener!’ Thus, rather than being unhelpfully imprecise, we suggest that the framework’s inherent fuzziness allows users space to interpret and adapt the six concepts in ways which make sense across an array of different contexts.

A second, probably more significant, limitation is that the framework has not been tested beyond England. Although the OECD tells us that CPDL contexts globally can be ‘difficult to navigate’ for schools and teachers, we simply do not know if the six concepts presented here would be meaningful or helpful to readers outside England. That said, we are confident that the issues explored here—formal and informal learning, individual and organisational learning, place, complexity, networks and so on—will resonate with many readers and that, if nothing else, the framework will provide a starting point for exploring the nature of coherence, quality and equity in their local learning landscapes.

## Conclusion

In conclusion, this article has argued that an appreciation of the continuing importance of place and the particular dynamics of local learning landscapes is important in decentralised education systems characterised by school autonomy, reduced local co-ordination mechanisms and a diverse array of professional development opportunities. These contexts might open up new possibilities for teachers and schools to learn, individually and collectively, but they can also be ‘difficult to navigate’ (Boeskens et al., 2020) and so present risks in terms of the coherence, quality and equity of CPDL opportunities and engagement. In England, we argue that the roll-back of LAs and the expansion of non-place-based MATs has led to a level of myopia in relation to the continuing importance of place as a space for professional learning. Furthermore, we argue that an exclusive focus on formal professional development programmes—by researchers and policy makers—risks accentuating this myopia, obscuring the extent to which teachers learn informally, within the context of their schools, networks and daily professional routines. The local learning landscapes framework set out here seeks to address these issues, informed by four different bodies of literature and refined through an iterative process of empirical research in England. In addition to setting out the framework and describing the development process and underpinning thinking, the article draws on the research in England to suggest that it makes three main contributions: as a heuristic device, an analytical tool and a methodological innovation. In the project report we explore some of the wider implications of the study for policy and practice in England. One clear conclusion is that strengthening coherence, quality and equity across local learning landscapes in this particular decentralised and fragmented system requires attention to system governance and design as well as leadership and locality dynamics.
